# IRES-Mediated Translation of Membrane Proteins and Glycoproteins in Eukaryotic Cell-Free Systems

**DOI:** 10.1371/journal.pone.0082234

**Published:** 2013-12-20

**Authors:** Andreas K. Brödel, Andrei Sonnabend, Lisa O. Roberts, Marlitt Stech, Doreen A. Wüstenhagen, Stefan Kubick

**Affiliations:** 1 Fraunhofer Institute for Biomedical Engineering (IBMT) Branch Potsdam-Golm, Potsdam, Germany; 2 Faculty of Health and Medical Sciences, University of Surrey, Guildford, United Kingdom; University of British Columbia, Canada

## Abstract

Internal ribosome entry site (IRES) elements found in the 5′ untranslated region of mRNAs enable translation initiation in a cap-independent manner, thereby representing an alternative to cap-dependent translation in cell-free protein expression systems. However, IRES function is largely species-dependent so their utility in cell-free systems from different species is rather limited. A promising approach to overcome these limitations would be the use of IRESs that are able to recruit components of the translation initiation apparatus from diverse origins. Here, we present a solution to this technical problem and describe the ability of a number of viral IRESs to direct efficient protein expression in different eukaryotic cell-free expression systems. The IRES from the intergenic region (IGR) of the Cricket paralysis virus (CrPV) genome was shown to function efficiently in four different cell-free systems based on lysates derived from cultured *Sf*21, CHO and K562 cells as well as wheat germ. Our results suggest that the CrPV IGR IRES-based expression vector is universally applicable for a broad range of eukaryotic cell lysates. *Sf*21, CHO and K562 cell-free expression systems are particularly promising platforms for the production of glycoproteins and membrane proteins since they contain endogenous microsomes that facilitate the incorporation of membrane-spanning proteins and the formation of post-translational modifications. We demonstrate the use of the CrPV IGR IRES-based expression vector for the enhanced synthesis of various target proteins including the glycoprotein erythropoietin and the membrane proteins heparin-binding EGF-like growth factor receptor as well as epidermal growth factor receptor in the above mentioned eukaryotic cell-free systems. CrPV IGR IRES-mediated translation will facilitate the development of novel eukaryotic cell-free expression platforms as well as the high-yield synthesis of desired proteins in already established systems.

## Introduction

Translation, the process of mRNA-encoded protein synthesis, can be subdivided into the phases of initiation, elongation, termination and ribosome recycling [1]. Of these phases, translation initiation is the most complex and rate-limiting step [2], representing a major barrier in the activation of a cell-free protein synthesis reaction [3]. In eukaryotes the initial primary transcript needs further processing before it becomes a functional mRNA on which translation initiation occurs [4], such as addition of a 5′ cap [5], [6]. However, capping of mRNA is a time-consuming and expensive production step in *in vitro* protein synthesis systems. Thus, recently developed eukaryotic cell-free expression systems have removed the requirement for this process by employing internal ribosome entry site (IRES) elements [7], [8], [9], [10]. IRES elements are highly structured RNA sequences found within the 5′ untranslated region (UTR) of viral genomes or cellular mRNAs that function to recruit ribosomes for the initiation of translation. IRESs direct translation of viral mRNAs and modulate translation of cellular mRNAs during development, stress, and disease, suggesting that these RNAs enable translation initiation when cap-dependent translation is downregulated [9]. Recognition of mRNAs by the cellular protein synthesis machinery is normally achieved through the binding of the 5′ cap by eIF4E, the cap-binding protein. This protein is one component of the cap-binding complex, eIF4F, which also includes eIF4A (an RNA helicase) and eIF4G, a scaffold protein which makes numerous contacts with other cellular proteins (including eIF3 and the poly(A) binding protein, PABP) and these interactions serve to bridge the gap between the mRNA and the small ribosomal subunit [11]. However, translation initiation on picornavirus RNAs occurs by a different mechanism. The 5′ UTR of all picornavirus genomes contains an IRES that directs cap-independent internal initiation of protein synthesis [12]. These IRESs range in size from tens to hundreds of nucleotides [13] and direct a cap-independent mechanism of translation initiation termed internal initiation [14], [15]. Several cellular RNA-binding proteins, named IRES trans-acting factors (ITAFs), have been identified that are required or at least enhance internal initiation [15], [16]. To date, the function of IRES elements has been shown to be largely species-dependent, although a small number have been shown to function across species [17], [18]. Therefore, many IRES elements do not initiate protein synthesis in cell extracts from various organisms. Recent advances have been made to overcome these limitations by designing a species-independent, universal translation-initiation leader that engages ribosomes directly, thereby bypassing the cap-dependent pathway [19]. Another promising approach to yield highly flexible templates is the utilization of cross-kingdom IRESs. These IRES elements have been reported to recruit components of the translation initiation apparatus from diverse origins [20].

In this study, we evaluated the ability of different viral IRES elements to initiate protein synthesis in various eukaryotic cell-free systems (wheat germ, *Sf*21, CHO and K562 cells). The efficiency of IRESs from the Encephalomyocarditis virus (EMCV), *Rhopalosiphum padi* virus (RhPV) and Cricket paralysis virus (CrPV) genomes were investigated. The main goal of the study was to identify IRESs that are universally applicable in a range of eukaryotic cell-free protein expression systems. From the IRESs tested, the IGR IRES from the CrPV genome was the most efficient across all cell-free systems. This result may facilitate the development of novel eukaryotic cell-free protein expression platforms as well as the high-yield synthesis of target proteins, in particular glycoproteins and membrane proteins, in already established *in vitro* transcription-translation systems. As a long-term goal, the optimization of cell-free systems shall facilitate the high-throughput synthesis of DNA libraries, the production of *in vivo* difficult-to-express proteins as well as the specific labeling of proteins. As glycoproteins and membrane proteins are of special pharmaceutical interest [21], [22], the work particularly highlights the cell-free expression of these types of proteins.

## Materials and Methods

### Materials

IRES sequences (EMCV and RhPV 5′) were present in the vector backbone pGEM-CAT/IRES/LUC (Promega, Mannheim, Germany). The RhPV 5′ IRES sequence used was the truncated version RhPVΔ1 as described previously [18]. In the case of the CrPV IGR IRES (GenBank accession no. AF218039, nucleotides 6025 to 6216), the complementary DNA (cDNA) was synthesized and cloned into the pMA vector backbone by Life Technologies (Darmstadt, Germany). IRES sequences used in this study are listed in Table 1 and illustrated in Figure S1. cDNAs encoding the fusion product of the enhanced yellow fluorescent protein (eYFP) and heparin-binding EGF-like growth factor (Hb-EGF-eYFP) as well as epidermal growth factor receptor (EGFR-eYFP), human erythropoietin (EPO) and firefly luciferase (LUC) were subcloned into the EasyXpress vectors pIX 2.0 and pIX 3.0 (Qiagen, Hilden, Germany), respectively. For this purpose, regulatory sequences (RS) were added at the 5′ and 3′ non-coding regions of the amplified gene of interests using the primers RS 5′ and RS 3′. The amplified templates were digested with suitable restriction nucleases and the resulting fragments were cloned into the EasyXpress pIX 2.0 or pIX 3.0 expression vector. For the constructs Mel-eYFP, Mel-Hb-EGF-eYFP, Mel-EPO and Mel-EGFR-eYFP, native signal peptides were replaced by the melittin signal sequence (Mel) or Mel was added to the N-terminus to enforce protein translocation into endogenous microsomes present in the applied eukaryotic cell lysates. EasyXpress constructs without IRES sequence were used as control plasmids to IRES-based expression.

**Table 1:** Summary of viral IRES elements investigated in this study.

Virus	Abbreviation	GenBank accession no.	IRES (nts of genome)
Encephalomyocarditis virus	EMCV	NC001479	273–833
*Rhopalosiphum padi* virus	RhPV 5′	NC001874	1–579
Cricket paralysis virus	CrPV IGR	NC003924	6025–6216

Numbering of IRES nucleotides corresponds to genomic position based on the sequence of the full-length genome.

Figure S1. IRES sequences are highlighted in

IGR: intergenic region.

### Generation of IRES-based expression vectors

Individual coding sequences were fused downstream of the cDNAs encoding IRESs by a three-step overlap extension (oe) PCR. In the first PCR step, individual cDNAs encoding the genes of interest and the IRES sequences were amplified (specific primers: CrPV IGR IRES-F, CrPV IGR IRES (GCT)-oe-EPO-R, CrPV IGR IRES (ATG)-oe-LUC-R, CrPV IGR IRES (GCT)-oe-LUC-R, CrPV IGR IRES (GCT)-oe-Mel-R, EMCV IRES-F, EMCV IRES-oe-LUC-R, LUC-F, LUC-R, RhPV 5′ IRES-F, RhPV 5′ IRES-oe-LUC-R, Table S1). The EMCV IRES was amplified in its truncated version from 273–836 bp. IGR IRES-mediated translation initiation does not depend on an AUG start codon [23]. In the case of the CrPV IGR IRES, the first translated amino acid is alanine encoded by a GCU base sequence [24], [25]. Accordingly, templates were constructed with an initiation codon AUG and an AUG-to-GCU mutation, thereby replacing the initial methionine by alanine [26]. In the second PCR step, IRES-specific forward and gene-specific reverse primer pairs were used to amplify and fuse individual IRESs to the gene of interest. Regulatory sequences containing the cloning sites EcoRI and XhoI were added at the 5′ and 3′ non-coding regions of the fusion constructs using the primers RS 5′ and RS 3′ (Table S1) in PCR step three. The amplified templates were digested with EcoRI and XhoI restriction nucleases and the resulting fragments were cloned into the EasyXpress pIX 3.0 expression vector in order to obtain the following constructs: pIX3.0-CrPV IGR IRES (GCT)-EPO, pIX3.0-CrPV IGR IRES (ATG)-LUC, pIX3.0-CrPV IGR IRES (GCT)-LUC, pIX3.0-CrPV IGR IRES (GCT)-Mel-eYFP, pIX3.0-CrPV IGR IRES (GCT)-Mel-Hb-EGF-eYFP, pIX3.0-EMCV IRES-LUC and pIX3.0-RhPV 5′ IRES-LUC. In the case of the pIX3.0-CrPV IGR IRES (GCT)-Mel-EGFR-eYFP, the CrPV IGR IRES sequence was removed from the constructed pIX3.0-CrPV IGR IRES (GCT)-Mel-eYFP vector by restriction digestion with Bst17ZI/NheI nucleases and the resulting fragment containing the CrPV IGR IRES was cloned into pIX3.0-Mel-EGFR-eYFP. Nucleotide sequences of all cloned constructs were confirmed by DNA sequencing. All plasmids are listed in Table S2 and a scheme of the different expression constructs is illustrated in Figure S2.

### Extract preparation procedure

#### Preparation of prokaryotic cell extracts

*Escherichia coli* (*E. coli*) lysates were prepared according to the method of Nirenberg [27] with slight modifications which have been described previously [28].

#### Preparation of eukaryotic cell extracts

Eukaryotic cells were grown exponentially in well-controlled fermenters. Insect cells (*Sf*21 cell line) were cultured at 27°C whereas suspension-adapted CHO and human K562 cells were grown at 37°C. Cell cultivations were performed using chemically defined, serum-free media (*Sf*21: Insect-XPRESS medium, Lonza; CHO: Power CHO-2 CD medium, Lonza; K562: ISF-1 serum-free medium, InVivo). Cells were harvested at a density of approximately 4.0×10^6^ cells/mL (*Sf*21), 2.0×10^6^ cells/mL (CHO) and 2.0×10^6^ cells/mL (K562) and collected by centrifugation at 200× g for 5 min. The resulting cell pellets were washed twice and resuspended with a buffer consisting of 40 mM HEPES-KOH (pH 7.5), 100 mM NaOAc and 4 mM DTT. Cells were disrupted mechanically by passing the cell suspension through a 20-gauge needle using a syringe. Next, the crude cell lysate was centrifuged at 10,000× g for 10 min in order to remove the nuclei and cell debris. Supernatants were applied to a Sephadex G-25 column (GE Healthcare, Freiburg, Germany), equilibrated with the above mentioned resuspension buffer, and the elution fractions (each 1 mL) with an RNA content above an absorbance of 100 at 260 nm were pooled. Cell lysates were treated with micrococcal nuclease (S7) in order to degrade residual mRNA. In this respect, 10 U/mL S7 nuclease (Roche, Mannheim, Germany) and 1 mM CaCl_2_ were added to the eluate and the reaction mixture was incubated for 2 min at room temperature. The reaction was inactivated by the addition of 6.7 mM EGTA (f. c.). Finally, cell lysates were immediately shock-frozen in liquid nitrogen and stored at −80°C to preserve maximum activity.

### Cell-free protein synthesis

Cell-free protein expression was performed in two different modes of transcription-translation procedures (linked and coupled batch reactions, Figure S3). In the linked system, transcription and translation reactions are separated by an intermediate gel filtration step (DyeEx spin columns, Qiagen) in order to purify the mRNA. *In vitro* transcription reactions were carried out for 2 h at 37°C using T7 RNA polymerase (Agilent, Waldbronn, Germany) as described previously [29], [30]. No cap analogue was added to the transcription reaction. RNA concentrations were measured using the Nanodrop 2000c spectrophotometer (Thermo Scientific). In contrast, the coupled system combines transcription and translation in a single batch reaction.

#### Prokaryotic cell-free protein synthesis

Coupled transcription-translation reactions were performed in 25 µL batch volumes for 90 min at 37°C in a thermomixer (Thermomixer comfort, Eppendorf, Hamburg, Germany) with shaking at 500 rpm. Reactions were composed of 35% (v/v) *E. coli* cell extract, 5 nM expression vector, 0.5 U/µL T7 RNA polymerase, nucleoside triphosphates (1.2 mM ATP, 0.8 mM CTP, 0.8 mM GTP and 0.8 mM UTP) and canonical amino acids, 1.2 mM each. To monitor protein quality and quantity, reaction mixtures were supplied with ^14^C-labeled leucine yielding a specific radioactivity of 1.5 dpm/pmol. The *in vitro* transcription-translation system based on *E. coli* lysate is commercially available (EasyXpress Protein Synthesis Kit, Qiagen).

#### Eukaryotic cell-free protein synthesis

Eukaryotic cell-free expression based on *Sf*21, CHO and K562 cell extracts was performed in linked as well as coupled reaction modes.

Linked translation reactions were composed of 40% (v/v) cell lysate, 200 µM of each canonical amino acid, 1.75 mM ATP, 0.45 mM GTP, 0.4 µM purified mRNA as well as 20 mM creatine phosphate and 100 µg/mL creatine kinase used for energy-regeneration. Translation reactions were performed in 25 µL batch volumes for 2 h in a thermomixer with shaking at 500 rpm and optimal reaction temperatures (*Sf*21 cell lysates, 27°C; CHO and K562 cell lysates, 30°C).

Coupled transcription-translation reactions in 25 µL batch volumes were composed of 40% (v/v) cell lysate, 100 µM of each canonical amino acid, nucleoside triphosphates (1.75 mM ATP, 0.30 mM CTP, 0.30 mM GTP and 0.30 mM UTP), 20–60 nM vector DNA, 1 U/µL T7 RNA polymerase, 20 mM creatine phosphate and 100 µg/mL creatine kinase. Protein expression was operated for 3 h in a thermomixer with shaking at 500 rpm and optimal reaction temperatures. Standard conditions were derived from initial data obtained for optimal cap-dependent transcription-translation reactions in the coupled cell-free system based on extracts from *Sf*21 cells. These standard conditions included the addition of 0.33 mM GpppG or 0.33 mM m^7^GpppG to the coupled *in vitro* reactions (Figure S4). In the case of CHO and K562 coupled cell-free expression, the incubation temperature was set to 30°C by default. These standard reaction conditions used in coupled reactions were adapted to CrPV IGR IRES-mediated translation (Table 2). To monitor protein quality, translation reaction mixtures were supplied with ^14^C-labeled leucine (specific radioactivity 75 dpm/pmol).

**Table 2:** Reaction conditions were adapted to CrPV IGR IRES-mediated translation in eukaryotic cell-free systems.

Coupled cell-free systems	*Sf*21 cell extract	CHO cell extract	K562 cell extract
Expression vector	60 nM/60 nM	60 nM/20 nM	60 nM/20 nM
KOAc	75 mM/120 mM	75 mM/150 mM	75 mM/120 mM
Mg(OAc)_2_	2.9 mM/3.4 mM	2.9 mM/3.9 mM	2.9 mM/3.4 mM
Incubation temperature	27°C/27°C	30°C/33°C	30°C/33°C

Reaction parameters used for cap-dependent translation in coupled reactions (left) were optimized for CrPV IGR IRES-mediated translation (right). Optimization was performed by cell-free expression of active LUC using the pIX3.0-CrPV IGR IRES (GCT) vector as a template. Reactions were incubated for 3 h in a thermomixer.

Eukaryotic cell-free protein expression based on wheat germ extract was performed in a continuous-exchange cell-free (CECF) system using the RTS 100 Wheat Germ CECF Kit (5 Prime, Hamburg, Germany) according to the manufacturer's protocol. Reactions were carried out for 24 h at 24°C.

### Determination of *de novo* synthesized active luciferase

Aliquots of 5 µL of the cell-free reaction mixtures were assayed for *de novo* synthesized active LUC (luminometer LB941, Berthold, Bad Wildbad, Germany) using LUC assay reagent (Promega, Mannheim, Germany). Relative light units were measured and the corresponding yields of active LUC in µg/mL were calculated based on a calibration curve.

### SDS-PAGE and autoradiography

Aliquots of cell-free reaction mixtures (7 µL) were subjected to cold acetone precipitation. After centrifugation (10 min, 16,000 g and 4°C), protein pellets were resuspended in 20 µL of 1× sample buffer (NuPAGE LDS Sample Buffer, Life Technologies) and loaded on a precast SDS-PAGE gel (NuPAGE 10% Bis-Tris gel, Life Technologies). Gels were run in MES SDS buffer for 35 min at 200 V. Subsequently, gels were dried for 70 min at 70°C (Unigeldryer 3545D, Uniequip, Planegg, Germany) and radioactively labeled proteins were visualized using the phosphorimager system (Typhoon TRIO+ Imager, GE Healthcare).

### Deglycosylation assay

Glycosylation of ^14^C-leucine-labeled, *de novo* synthesized EPO was investigated by PNGase F (NEB, Frankfurt, Germany) treatment of a 7 µL aliquot of the K562 cell-free reaction mixture. Samples were treated according to the manufacturer's instructions. Translation mixtures (TMs) were loaded on SDS-PAGE gels before and after deglycosylation of radiolabeled, *de novo* synthesized EPO and samples were subsequently analyzed by autoradiography.

### Fluorescence analysis

Translocation of Mel-eYFP, Mel-Hb-EGF-eYFP and Mel-EGFR-eYFP fusion proteins into microsomal membranes was visualized by confocal laser scanning microscopy (LSM 510, Zeiss, Jena, Germany) and analyzed by Zeiss LSM Imaging software. Samples were diluted 1∶1 in phosphate buffered saline (PBS without Mg^2+^ and Ca^2+^, Biochrom AG), transferred to ibidi slides (μ-slide, 18 well, Ibidi, Planegg, Germany) and eYFP was excited at 488 nm using an argon laser. Fluorescence emission was recorded with a long-pass filter in the wavelength range above 505 nm. For the analysis of the expression levels of the fusion proteins, eYFP was excited at 488 nm and fluorescence signals were recorded at 526 nm using the Typhoon TRIO+ Imager. Obtained images were analyzed using the ImageQuant TL software. No template controls were prepared in the same way as the samples with the exception of the DNA template, which was replaced by RNase-free water.

## Results

### Efficiency of IRES-mediated translation in different cell-free lysates

**Figure 1:**
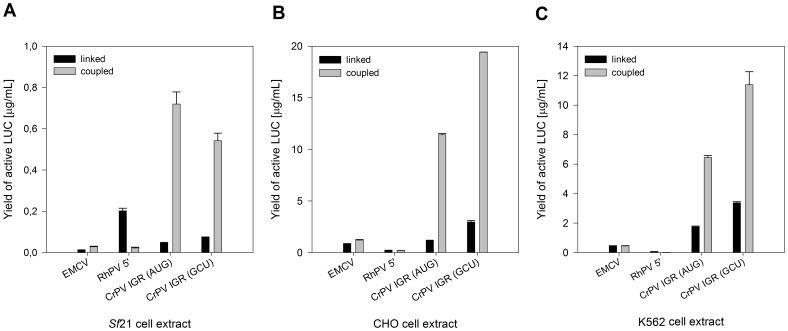
IRES-mediated translation in eukaryotic cell-free systems. LUC encoding expression constructs harboring different IRESs in the EasyXpress pIX3.0 vector backbone were investigated. Cell-free protein synthesis was performed in linked and coupled transcription-translation systems using (A) *Sf*21, (B) CHO and (C) K562 cell extracts. Vectors containing a specific IRES are indicated by the name of the IRES insert. In the case of the CrPV IGR IRES, the influence of an AUG-to-GCU mutation of the initiation codon was investigated. Reactions were performed at standard conditions in the absence of a cap analogue. Relative light units were measured using a LUC reporter assay and the corresponding yields of active LUC in µg/mL were calculated based on a calibration curve. Yields of active LUC were determined from three independent experiments and the corresponding standard deviations were calculated.

Protein expression levels of different LUC encoding vectors harboring IRESs in the EasyXpress pIX3.0 vector backbone were investigated in *Sf*21, CHO and K562 linked and coupled cell-free systems after 3 h of incubation (Figure 1). The coupled system combined transcription and translation in one reaction compartment. In contrast, transcription and translation reactions were separated by an intermediate gel filtration step in the linked system. Reactions were performed at standard conditions in the absence of a cap analogue.

Yields of active LUC were dependent on the investigated IRES in each cell-free system. Highest yields of *de novo* synthesized LUC were obtained from expression constructs containing the CrPV IGR IRES in coupled cell-free platforms. Replacement of the initiator AUG-codon to a GCU-codon resulted in a further improvement of protein expression levels in each cell-free system except for coupled *Sf*21 cell lysate-based protein synthesis. The CrPV IGR IRES-mediated translation led to 0.7 µg/mL (*Sf*21 cell lysate), 19.4 µg/mL (CHO cell lysate) and 11.4 µg/mL (K562 cell lysate) of *de novo* synthesized active LUC.

### Optimization of CrPV IGR IRES-mediated translation

**Figure 2:**
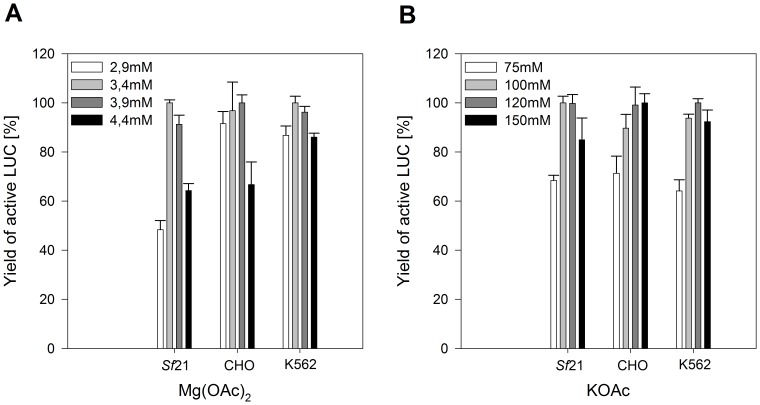
Evaluation of optimal Mg(OAc)_2_ (A) and KOAc (B) concentration in coupled eukaryotic cell-free expression systems. *De novo* synthesized LUC was monitored after 3 h of incubation at 27°C (*Sf*21 cell extract) and 33°C (CHO and K562 cell extracts), respectively. Cell-free protein synthesis was performed using the optimized vector containing the CrPV IGR IRES harboring an AUG-to-GCU mutation of the initiation codon in the EasyXpress pIX3.0 vector backbone. Protein yields were normalized to the reaction with the highest yield of active LUC ( = 100%) for each cell-free system. Yields of active LUC were determined from three independent experiments using a LUC reporter assay and the corresponding standard deviations were calculated.

Reaction temperature, incubation time, ion concentrations (Mg^2+^ and K^+^) and vector concentration were adapted to CrPV IGR IRES-mediated translation of target proteins in coupled cell-free systems based on lysates from cultured *Sf*21, CHO and K562 cells. The LUC encoding expression construct containing the CrPV IGR IRES harboring the AUG-to-GCU mutation of the initiation codon in the EasyXpress pIX3.0 backbone (pIX3.0-CrPV IGR IRES (GCT)-LUC) was used for the optimization of reaction conditions. *De novo* synthesized active LUC was monitored after 3 h of incubation and reactions with the highest yields of active LUC were normalized to 100% (Figure 2). Highest yields of active LUC were obtained at final Mg(OAc)_2_ concentrations in the range of 3.4 mM to 3.9 mM (Figure 2A). Maximum expression levels of active LUC were observed at final KOAc concentrations in the range of 100 mM to 150 mM (Figure 2B). Similar experiments were performed for all of the above mentioned reaction parameters in coupled *Sf*21, CHO and K562 cell-free platforms (data not shown). Standard and optimized reaction conditions are listed in Table 2.

**Figure 3:**
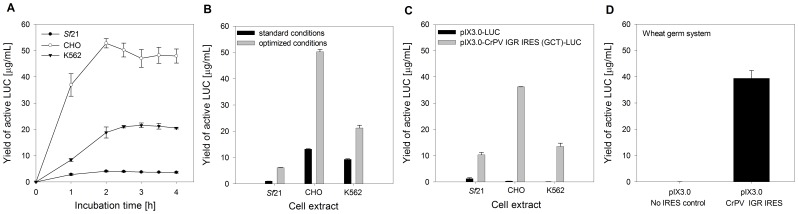
CrPV IGR IRES-mediated translation in coupled eukaryotic cell-free expression systems. A) Time course analysis of cell-free synthesized LUC within 4 h of incubation using optimized reaction conditions. B) Impact of the optimization process on CrPV IGR IRES-mediated protein synthesis. Reaction temperature, incubation time, template concentration and ion concentrations (Mg(OAc)_2_ and KOAc) were adapted to CrPV IGR IRES-directed translation. C) Comparison of the IRES-independent translation at standard conditions and CrPV IGR IRES-dependent translation at optimized conditions in coupled eukaryotic cell-free systems. Translations were carried out in the presence of 0.33 mM m^7^GpppG cap analogue. D) CrPV IGR IRES-mediated translation in a wheat germ CECF system within 24 h of incubation at 24°C. 100 mM KOAc was added to each dialysis reaction. Relative light units were measured using a LUC reporter assay and the corresponding yields of active LUC in µg/mL were calculated based on a calibration curve. Yields of active LUC were determined from three independent experiments and the corresponding standard deviations were calculated.

The initial optimization process was followed by an evaluation of the individual cell-free system's productivity. A time course analysis of cell-free expressed active LUC was performed within 4 h of incubation (Figure 3A). *In vitro* translation using optimized CrPV IGR IRES constructs led to 4.1 µg/mL (*Sf*21 cell lysate), 52.8 µg/mL (CHO cell lysate) and 21.5 µg/mL (K562 cell lysate) of *de novo* synthesized active LUC. Maximum protein yields were obtained after 2 h of incubation for the CHO and *Sf*21 cell lysates and 3 h when using K562 cell extracts. Extended reaction times did not further increase the yield of cell-free expressed and active LUC. An analysis of *de novo* synthesized LUC in eukaryotic cell-free systems when using standard conditions compared to optimized reaction conditions is illustrated in Figure 3B. Adaptation of reaction conditions to CrPV IGR IRES-mediated translation significantly upregulated the synthesis of active LUC in each eukaryotic cell-free system. Expression levels of active target protein shifted from initially 1.0 µg/mL to 6.1 µg/mL (*Sf*21 cell lysate), 13.2 µg/mL to 50.2 µg/mL (CHO cell lysate) and 9.3 µg/mL to 21.2 µg/mL (K562 cell lysate), thus resulting in a two to six fold increase of *de novo* synthesized active LUC compared to the expression in cell-free systems which have not been adapted to the CrPV IGR IRES. In addition, a comparison of the IRES-independent translation at standard conditions and the CrPV IGR IRES-dependent translation at optimized conditions in coupled eukaryotic cell-free systems was performed (Figure 3C). Translations were carried out in the presence of 0.33 mM m^7^GpppG cap analogue. Protein expression levels were significantly higher using the IRES-based compared to the non-IRES-based translation in each cell-free system. IRES-independent translation yielded 1.2 µg/mL (*Sf*21 cell lysate), 0.3 µg/mL (CHO cell lysate) and 0.1 µg/mL (K562 cell lysate) of *de novo* synthesized active LUC. In contrast, *in vitro* translation using the CrPV IGR IRES construct led to 10.3 µg/mL (*Sf*21 cell lysate), 36.2 µg/mL (CHO cell lysate) and 13.5 µg/mL (K562 cell lysate) of *de novo* synthesized active LUC. As a result of different lysate batches, yields of active LUC obtained from CrPV IGR IRES-dependent translation slightly varied in Figure 3B and Figure 3C.

CrPV IGR IRES-mediated translation in plant-derived cell-free platforms was investigated by measuring LUC expression in a wheat germ-based CECF system (Figure 3D). KOAc concentration was initially adapted to CrPV IGR IRES-directed translation (Figure S5), resulting in an addition of 100 mM KOAc to each dialysis reaction. The LUC encoding EasyXpress pIX3.0 vector was used as a control template to demonstrate the impact of the CrPV IGR IRES on LUC expression. 39.4 µg/mL *de novo* synthesized active LUC was synthesized from the CrPV IGR IRES after 24 h of incubation compared to 0.02 µg/mL active target protein using the control plasmid without IRES.

### Impact of CrPV IGR IRES-mediated translation initiation on protein expression levels

**Figure 4:**
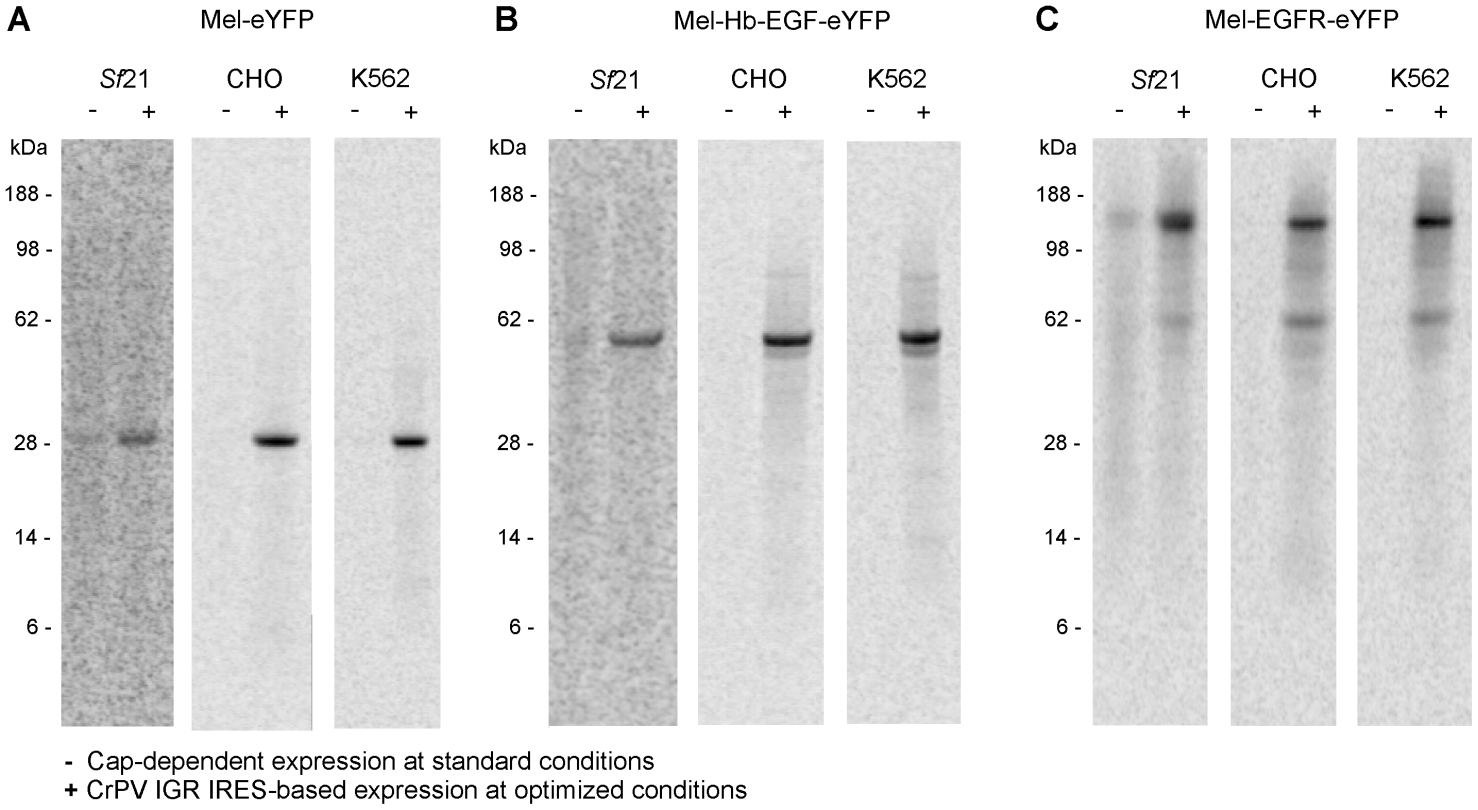
Autoradiographic analysis of CrPV IGR IRES-mediated translation in coupled eukaryotic cell-free systems. The influence of the CrPV IGR IRES on protein expression of the secreted protein Mel-eYFP (29 kDa; A) as well as the membrane proteins Mel-Hb-EGF-eYFP (51 kDa; B) and Mel-EGFR-eYFP (162 kDa; C) was investigated in coupled systems based on lysates from *Sf*21, CHO and K562 cells. Plasmids encoding the target protein were equipped with (+) or without (−) the CrPV IGR IRES (GCU) in the EasyXpress pIX3.0 vector backbone. ^14^C-leucine-labeled, *de novo* synthesized proteins were visualized by autoradiography after gel electrophoresis.

The impact of CrPV IGR IRES-mediated translation initiation on protein expression levels was investigated by analyzing the synthesis of the secreted protein Mel-eYFP (29 kDa) as well as type I transmembrane proteins heparin-binding EGF-like growth factor (Hb-EGF) and epidermal growth factor receptor (EGFR). The native signal peptides of the membrane proteins were replaced by the melittin signal sequence and eYFP was fused C-terminally to the encoded protein to facilitate the analysis of protein localization by fluorescence microscopy (Mel-Hb-EGF-eYFP, 51 kDa and Mel-EGFR-eYFP, 162 kDa). The EasyXpress pIX3.0 vector backbone was used as control template to demonstrate the influence of the CrPV IGR IRES upstream of the target protein encoding sequence on protein expression. CrPV IGR IRES-mediated cell-free reactions were performed at optimized conditions whereas pIX3.0-based expression of the target proteins was operated at reaction conditions used for cap-dependent translation by default. In this context, reactions were supplemented with 0.33 mM GpppG cap analogue. The unmethylated cap was used instead of the methylated form as it is significantly cheaper. The use of the methylated cap results in slightly increased expression levels in the coupled systems (Figure S4). Cell-free expressed and ^14^C-leucine-labeled proteins were visualized by autoradiography after gel electrophoresis (Figure 4). All target proteins were synthesized in each eukaryotic cell-free system containing the CrPV IGR IRES-based expression vectors. Full-length Mel-eYFP, Mel-Hb-EGF-eYFP and Mel-EGFR-eYFP were observed at the expected molecular mass of 29 kDa, 51 kDa and 162 kDa, respectively. Mel-eYFP was detected without any signs of proteolysis or fragmentation in all analyzed cell-free systems whereas synthesized Mel-Hb-EGF-eYFP using CHO and K562 extracts resulted in an additional band at a slightly lower apparent molecular mass. Mel-EGFR-eYFP expression resulted in the full-length protein and additional bands each migrating at a lower apparent molecular mass. A significantly upregulated expression of all target proteins was observed from the CrPV IGR IRES containing constructs compared to the control reactions in each of the investigated cell-free systems. *De novo* synthesized target proteins could not be detected when performing EasyXpress pIX3.0-based control reactions in CHO and K562 cell extracts.

**Figure 5:**
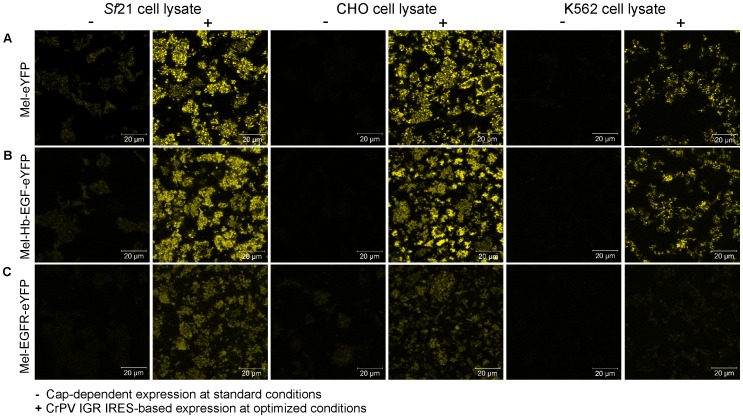
CLSM analysis of eYFP-tagged proteins synthesized in coupled *Sf*21, CHO and K562 cell-free expression systems. CLSM images depict the *de novo* synthesized secreted protein Mel-eYFP (A) as well as the membrane proteins Mel-Hb-EGF-eYFP (B) and Mel-EGFR-eYFP (C). Plasmids encoding the target proteins were equipped with (+) or without (−) the CrPV IGR IRES (GCU). In the case of Mel-eYFP, fluorescent vesicles indicate the translocation of the target protein into the lumen of the endogenous microsomes present in cell-free systems based on cultured *Sf*21, CHO and K562 cells. In the case of Mel-Hb-EGF-eYFP and Mel-EGFR-eYFP, microsomes show a fluorescent membrane due to the insertion of *de novo* synthesized membrane proteins. eYFP was excited at 488 nm and fluorescence emission was recorded with a long-pass filter in the wavelength range above 505 nm (LSM 510 Meta microscope, Zeiss).

**Figure 6:**
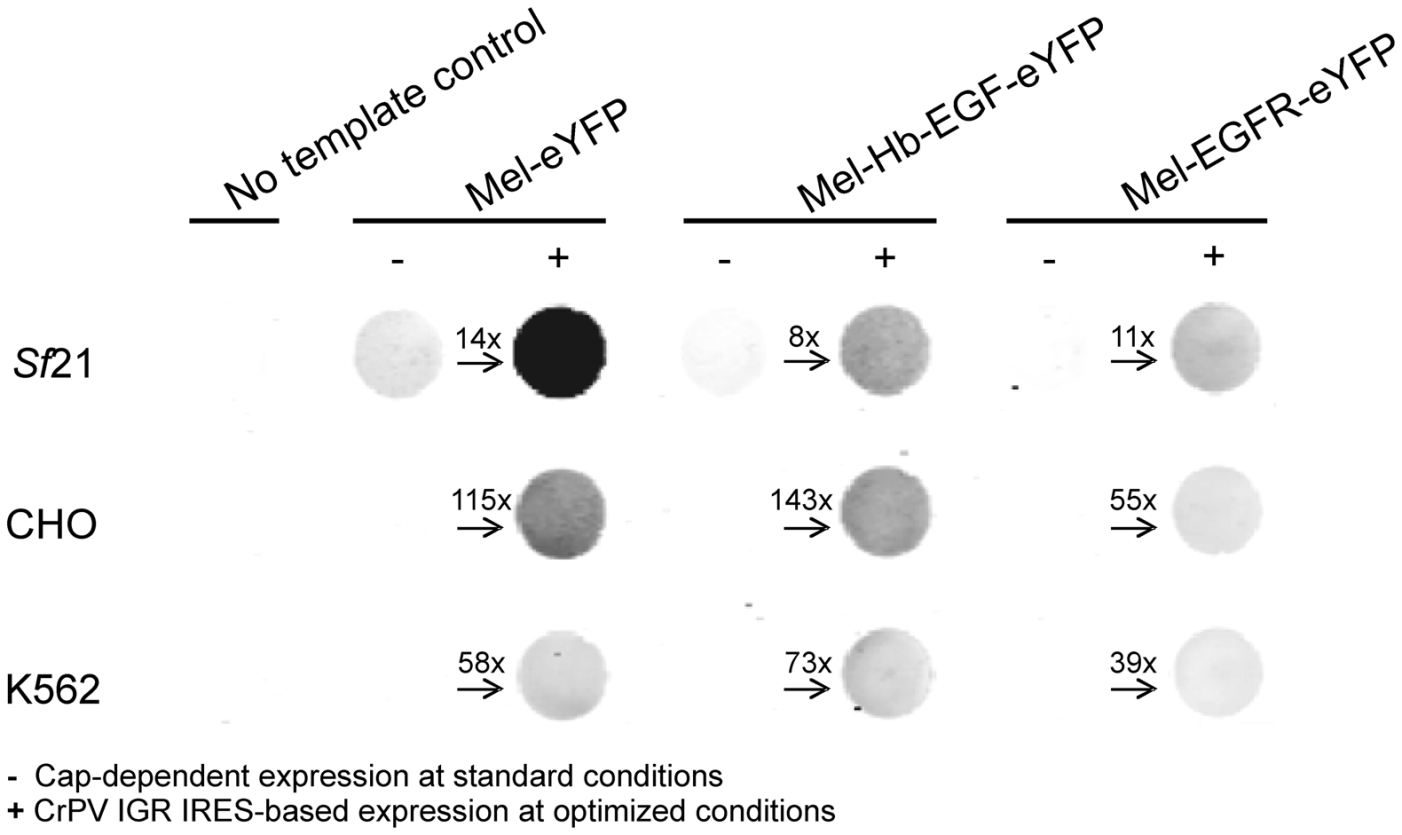
Fluorescence analysis of eYFP-tagged proteins synthesized in coupled *Sf*21, CHO and K562 cell-free expression systems. The image depicts the *de novo* synthesized secreted protein Mel-eYFP as well as the membrane proteins Mel-Hb-EGF-eYFP and Mel-EGFR-eYFP. Plasmids encoding the target proteins were equipped with (+) or without (−) the CrPV IGR IRES (GCU). Numbers indicate the increase of the expression levels using the CrPV IGR IRES-based construct compared to the no IRES control. No template controls were prepared in the same way as the samples, but instead of a DNA template, RNase-free water was added to the reaction. Samples were analyzed using the phosphorimager system (Typhoon TRIO+ Imager, GE Healthcare).

Autoradiographic data were complemented by fluorescence microscopy of the above mentioned *de novo* synthesized fusion proteins (Figure 5). CLSM images were recorded with the same microscopic settings for each protein of interest in the respective *in vitro* system to investigate the influence of CrPV IGR IRES-mediated translation on protein expression and localization by measuring the expressed protein's fluorescence intensities. Due to varying signal intensities, CLSM settings differed between different cell-free systems and thus the fluorescence intensities cannot be compared among *in vitro* platforms. CLSM images of samples harboring *de novo* synthesized Mel-eYFP depicted fluorescent microsomes, thus indicating translocation and incorporation of the target protein into the lumen of the microsomal vesicles present in *Sf*21, CHO and K562 cell-free systems (Figure 5A). In the case of cell-free expressed fluorescently labeled membrane proteins, microsomes show a fluorescent membrane due to the insertion of *de novo* synthesized membrane-spanning proteins Mel-Hb-EGF-eYFP and Mel-EGFR-eYFP, respectively (Figure 5B and C, Figure S6). In each of the investigated eukaryotic cell-free systems CrPV IGR IRES-directed translation of all target proteins resulted in significantly increased fluorescence intensities when compared to the control reactions without an IRES, except for Mel-EGFR-eYFP synthesized in the K562 cell lysates. Additionally, fluorescence intensities of eYFP-tagged proteins were investigated using the phosphorimager system in order to quantitatively analyze the increase of the CrPV IGR IRES-mediated translation compared to the no IRES control (Figure 6). Expression levels of Mel-eYFP increased by the factor of 14 (*Sf*21 cell lysate), 115 (CHO cell lysate) and 58 (K562 cell lysate). CrPV IGR IRES-directed translation of Mel-Hb-EGF-eYFP resulted in an 8-fold (*Sf*21 cell lysate), 143-fold (CHO cell lysate) and 73-fold (K562 cell lysate) increase compared to the control. Expression levels of Mel-EGFR-eYFP increased by the factor of 11 (*Sf*21 cell lysate), 55 (CHO cell lysate) and 39 (K562 cell lysate) using the CrPV IGR IRES-based construct compared to the no IRES control.

### CrPV IGR IRES-mediated translation of glycoproteins

**Figure 7:**
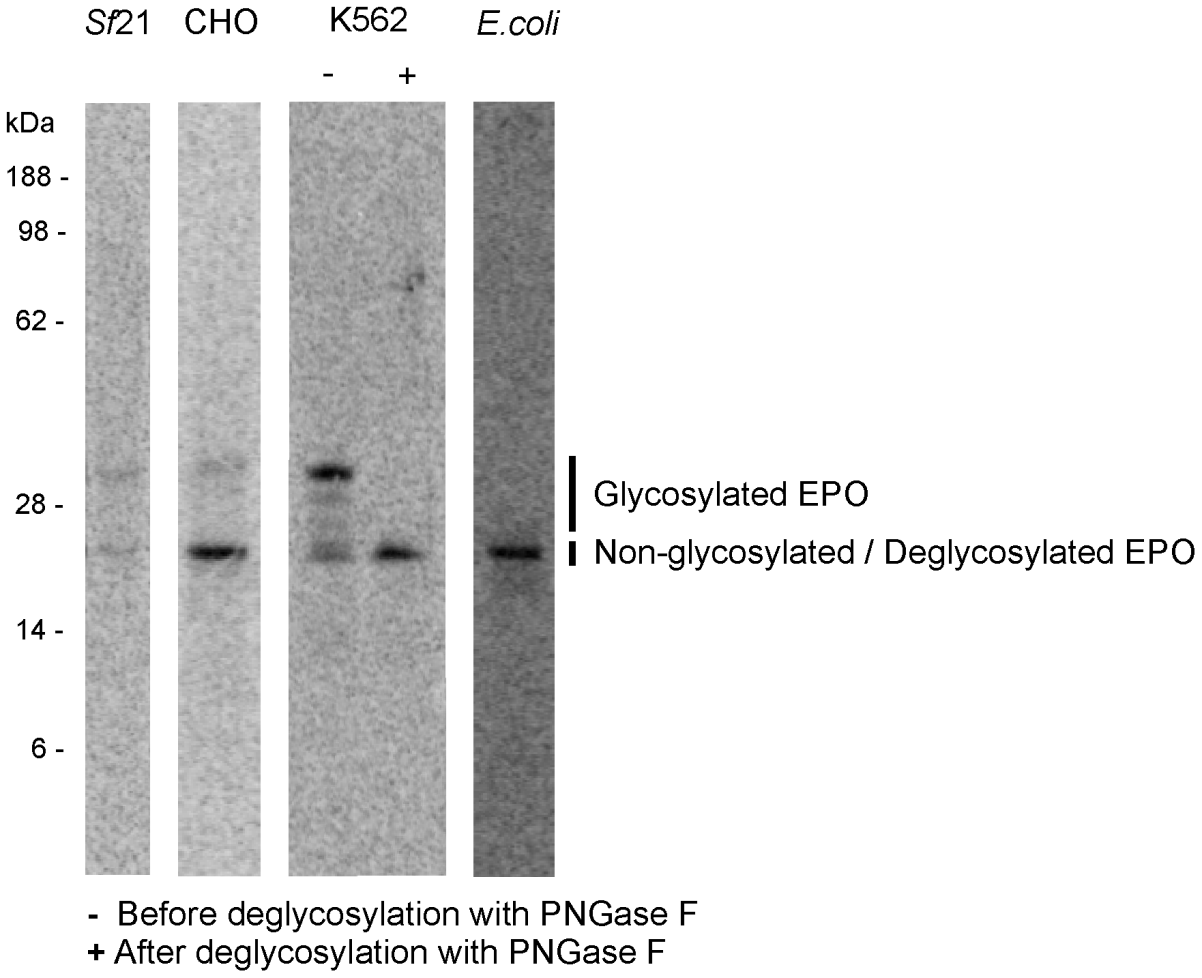
Expression of the glycoprotein EPO in prokaryotic and eukaryotic cell-free systems. Eukaryotic expression was performed using the EasyXpress pIX3.0 vector equipped with the CrPV IGR IRES (GCU). In the case of the K562-based cell-free expression, EPO is presented before (−) and after (+) deglycosylation with PNGase F. For prokaryotic expression, the native signal peptide of EPO was replaced by the melittin signal sequence and cell-free expression of the target protein was operated using the EasyXpress pIX2.0 vector. ^14^C-leucine-labeled, *de novo* synthesized EPO (21 kDa, non-glycosylated) was visualized by autoradiography after gel electrophoresis.

CrPV IGR IRES-mediated translation of glycoproteins was studied by the analysis of cell-free expression of human erythropoietin (EPO). The target protein harbors three N-linked glycosylation sites and migrates on SDS-PAGE gels at an apparent molecular mass of 21 kDa in its unprocessed form [31]. A comparative analysis of cell-free expressed EPO in batch-based prokaryotic and eukaryotic cell-free systems was performed. The pIX3.0-CrPV IGR IRES-based constructs, used in this study, are generally non-functional in prokaryotic cell-free systems as the eukaryotic ribosomal protein RPS25 is essential for IGR IRES activity [32]. Therefore, the EasyXpress pIX2.0-Mel-EPO vector, which harbors a Shine-Dalgarno sequence immediately upstream of the gene's open reading frame, was used as the plasmid of choice for the expression of EPO in the *E. coli*-based cell-free protein expression system. ^14^C-leucine-labeled, *de novo* synthesized EPO was visualized by autoradiography (Figure 7). As expected, *E. coli*-based cell-free expression of EPO resulted in one apparent band migrating at 21 kDa thus showing no glycosylation of the target protein. In contrast, *in vitro* translation of EPO in *Sf*21, CHO and K562-based systems resulted in additional bands migrating between 21 kDa and 32 kDa. To confirm that the migration process of the bands above 21 kDa was due to glycosylation of the target protein, cell-free synthesized EPO was treated with PNGase F, an enzyme which removes N-linked glycans. After PNGase F treatment of EPO synthesized in the K562 cell extract, the upper bands were converted into a single band migrating at a lower apparent molecular mass, thus demonstrating the specific cleavage of sugar moieties that have been attached to the glycoprotein during cell-free protein synthesis.

## Discussion

In this study, the efficiency of IRES-mediated translation has been investigated in order to facilitate the construction of a universally applicable vector for the synthesis of target proteins, including membrane proteins and glycoproteins, in eukaryotic cell-free expression systems. Out of the three investigated IRESs, highest yields of *de novo* synthesized LUC were obtained from expression constructs containing the CrPV IGR IRES in each of the investigated coupled cell-free systems. As it is known that the majority of Dicistrovirus IGR IRESs display maximum efficiency when GCU is the initiation codon [26], we assessed the efficiency of this IRES in this context compared to an AUG codon-containing IRES. Replacement of the initiation codon AUG to GCU further increased protein expression levels in coupled CHO and K562 cell-free systems.

Optimum reaction conditions for CrPV IGR IRES-mediated translation differ from standard conditions used for cap-dependent translation [17]. Protein expression levels from the CrPV IGR IRES-containing construct were upregulated compared to the standard conditions by adapting the reaction conditions, yielding 6 µg/mL (*Sf*21 lysates), 50 µg/mL (CHO lysates) and 21 µg/mL (K562 lysates) of active LUC. These results illustrate a two to six fold increase of *de novo* synthesized active LUC compared to the expression in non-CrPV IGR IRES-adapted cell-free systems. The difference in protein expression levels among cell extracts derived from diverse species is caused by different performances of the cell-free systems. The translational activity of a cell extract is one important factor that affects the *in vitro* system's performance. Another critical point to consider is the activity of the CrPV IGR IRES, which is known to be dependent on the specific eukaryotic species [26]. In addition, diverse transcription rates in the coupled systems, which result in different mRNA concentrations, may also affect the *in vitro* system's performance. The efficiency of the CrPV IGR IRES-based construct was also demonstrated in a plant-derived cell-free system as most of the mammalian virus IRESs do not function in plants [33]. However, the CrPV IGR IRES efficiently initiates translation in wheat germ extracts [24], demonstrating the use of the CrPV IGR IRES-based vector for protein expression in plant-derived cell-free systems. Since the CrPV IGR IRES has also been reported to promote translation in yeast strains [34], [35], the CrPV IGR IRES-based vector is suggested as a universally applicable construct for eukaryotic cell-free protein expression.

The ability of the CrPV IGR IRES to function in various eukaryotic cell-free systems is probably due to its unique structure. Translation in eukaryotes is mainly regulated at the initiation step [2], [36] and the CrPV IGR IRES promotes translation initiation in the absence of any initiation factors [23], [35], [37]. Accordingly, the CrPV IGR IRES bypasses all the steps of conventional eukaryotic translation initiation [25], [38], [39] which are known as major bottlenecks in cell-free protein expression [3], [36], [40]. Furthermore, as the CrPV IGR IRES directly interacts with the ribosome, which tends to be more conserved than the translation initiation factors, CrPV IGR IRES-mediated translation is probably less species-dependent than regulatory sequences that need to bind to initiation factors. In this way, the CrPV IGR IRES enables protein expression in a range of eukaryotic cell extracts.

The performance of the eukaryotic CrPV IGR IRES-based expression vector has been evaluated for its ability to direct synthesis of a variety of target proteins, including glycoproteins and membrane proteins. The data obtained demonstrated significantly upregulated expression of the secreted protein Mel-eYFP as well as the membrane proteins Mel-Hb-EGF-eYFP and Mel-EGFR-eYFP in each of the investigated eukaryotic cell-free systems by CrPV IGR IRES-mediated translation compared to control reactions with vectors without an IRES sequence. It has to be mentioned that the impact of the CrPV IGR IRES on protein expression levels varied between different target proteins in each cell-free system. Eukaryotic cell lysates harbor ER-derived microsomes allowing for the embedding of membrane proteins in their membranes and storage of *de novo* synthesized secretory proteins in their lumen [41], [42], [43]. Fluorescent microsomes that have been visualized by CLSM analysis indicate the expression, translocation and incorporation of Mel-eYFP into the lumen of endogenous vesicles present in cell-free systems based on cultured *Sf*21, CHO and K562 cells. In the case of *de novo* synthesized Mel-Hb-EGF-eYFP and Mel-EGFR-eYFP, microsomes show a fluorescent membrane due to insertion of the target proteins in the membrane of microsomal vesicles. Significantly improved protein expression levels have been obtained from CrPV IGR IRES-based constructs compared to control reactions in the above mentioned eukaryotic cell-free systems, proving the impact of the utility of the CrPV IGR IRES on production of translocated proteins.

N-linked glycosylation influences protein function, stability and protein complex formation in eukaryotes, demonstrating the pharmacological relevance of glycoprotein synthesis [44], [45]. The use of the CrPV IGR IRES-based expression vector for the synthesis of glycosylated proteins in various eukaryotic *in vitro* translation systems has been investigated by the expression of the human glycoprotein EPO using cell extracts derived from cultured *Sf*21, CHO and K562 cells. Autoradiographic data demonstrated the CrPV IGR IRES-mediated cell-free synthesis of N-glycosylated EPO in eukaryotic cell-free systems.

The CrPV IGR IRES-based expression vectors described here may facilitate the improved synthesis of target proteins including glycoproteins and membrane proteins in a variety of eukaryotic cell-free systems. CrPV IGR IRES-mediated translation could facilitate the development of novel eukaryotic cell-free platforms as well as the high-yield expression of target proteins in already established eukaryotic systems.

## Supporting Information

Figure S1 **IRES sequences used in this study.**
(DOCX)

Figure S2 **Scheme of the expression constructs used in this study.**
(DOCX)

Figure S3 **Workflow of the cell-free protein synthesis reactions using eukaryotic cell extracts.**
(DOCX)

Figure S4 **Impact of the cap structure on the expression levels in coupled eukaryotic cell-free systems.**
(DOCX)

Figure S5 **Evaluation of optimal KOAc concentration in the wheat germ-based CECF system.**
(DOCX)

Figure S6 **Integration of membrane proteins into microsomal vesicles.**
(DOCX)

Table S1 **Primer sequences used for DNA template design.**
(DOCX)

Table S2 **Expression vectors used in this study.**
(DOCX)
