# Supplementary material for: IRES-Mediated Translation of Membrane Proteins and Glycoproteins in Eukaryotic Cell-Free Systems
Source: PLoS One. 2013 Dec 20;8(12):e82234. doi: 10.1371/journal.pone.0082234 (PMC3869664; doi:10.1371/journal.pone.0082234)
Supplement: Figure S1 — IRES sequences used in this study. (DOCX) [file pone.0082234.s001.docx]

**A**

EMCV IRES

  1 CCCCCCCCTA ACGTTACTGG CCGAAGCCGC TTGGAATAAG GCCGGTGTGC GTTTGTCTAT 
 61 ATGTTATTTT CCACCATATT GCCGTCTTTT GGCAATGTGA GGGCCCGGAA ACCTGGCCCT 
121 GTCTTCTTGA CGAGCATTCC TAGGGGTCTT TCCCCTCTCG CCAAAGGAAT GCAAGGTCTG 
181 TTGAATGTCG TGAAGGAAGC AGTTCCTCTG GAAGCTTCTT GAAGACAAAC AACGTCTGTA 
241 GCGACCCTTT GCAGGCAGCG GAACCCCCCA CCTGGCGACA GGTGCCTCTG CGGCCAAAAG 
301 CCACGTGTAT AAGATACACC TGCAAAGGCG GCACAACCCC AGTGCCACGT TGTGAGTTGG 
361 ATAGTTGTGG AAAGAGTCAA ATGGCTCTCC TCAAGCGTAT TCAACAAGGG GCTGAAGGAT 
421 GCCCAGAAGG TACCCCATTG TATGGGATCT GATCTGGGGC CTCGGTGCAC ATGCTTTACA 
481 TGTGTTTAGT CGAGGTTAAA AAAACGTCTA GGCCCCCCGA ACCACGGGGA CGTGGTTTTC 
541 CTTTGAAAAA CACGATGATA AT

**B**

RhPV 5‘ IRES

  1 GATAAAAGAA CCTATAATCC CTTCGCACAC CGCGTCACAC CGCGCTATAT GCTGCTCATT 
 61 AGGAATTACG GCTCCTTTTT TGTGGATACA ATCTCTTGTA TACGATATAC TTATTGTTAA 
121 TTTCATTGAC CTTTACGCAA TCCTGCGTAA ATGCTGGTAT AGGGTGTACT TCGGATTTCC 
181 GAGCCTATAT TGGTTTTGAA AGGACCTTTA AGTCCCTACT ATACTACATT GTACTAGCGT 
241 AGGCCACGTA GGCCCGTAAG ATATTATAAC TATTTTATTA TATTTTATTC ACCCCCCACA 
301 TTAATCCCAG TTAAAGCTTT ATAACTATAA GTAAGCCGTG CCGAAACGTT AATCGGTCGC 
361 TAGTTGCGTA ACAACTGTTA GTTTAATTTT CCAAAATTTA TTTTTCACAA TTTTTAGTTA 
421 AGATTTTAGC TTGCCTTAAG CAGTCTTTAT ATCTTCTGTA TATTATTTTA AAGTTTATAG 
481 GAGCAAAGTT CGCTTTACTC GCAATAGCTA TTTTATTTAT TTTAGGAATA TTATCACCTC 
541 GTAATTATTT AATTATAACA TTAGCTTTAT CTATTTATA

**C**

CrPV IGR IRES

  1 AAAGCAAAAA TGTGATCTTG CTTGTAAATA CAATTTTGAG AGGTTAATAA ATTACAAGTA 
 61 GTGCTATTTT TGTATTTAGG TTAGCTATTT AGCTTTACGT TCCAGGATGC CTAGTGGCAG 
121 CCCCACAATA TCCAGGAAGC CCTCTCTGCG GTTTTTCAGA TTAGGTAGTC GAAAAACCTA 
181 AGAAATTTAC CT

**Figure S1. IRES sequences used in this study.**
