# Supplementary material for: IRES-Mediated Translation of Membrane Proteins and Glycoproteins in Eukaryotic Cell-Free Systems
Source: PLoS One. 2013 Dec 20;8(12):e82234. doi: 10.1371/journal.pone.0082234 (PMC3869664; doi:10.1371/journal.pone.0082234)
Supplement: Figure S2 — Scheme of the expression constructs used in this study. (DOCX) [file pone.0082234.s002.docx]

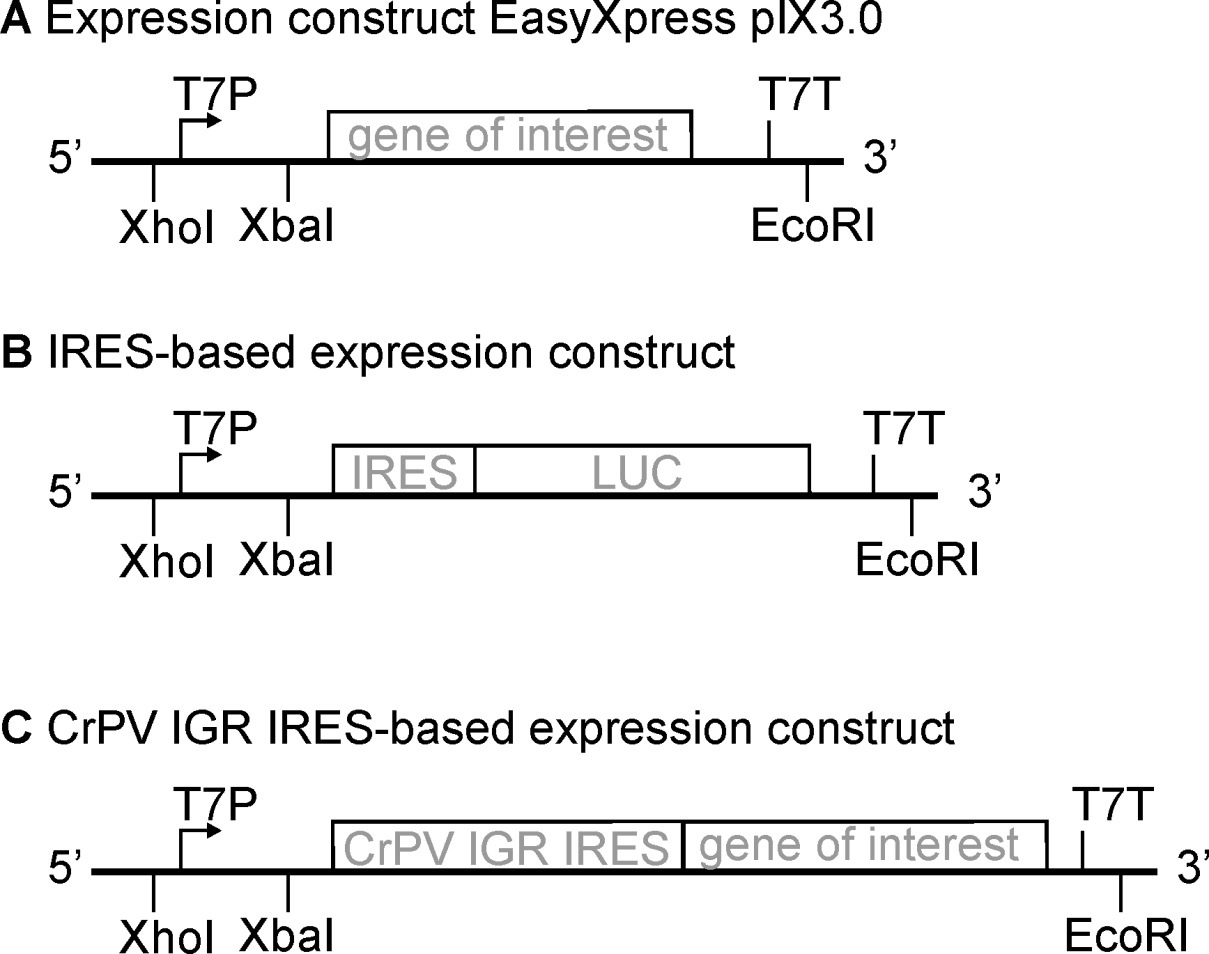


**Figure S2. Scheme of the expression constructs used in this study.** A) The construct EasyXpress pIX3.0 was used as a control to CrPV IGR IRES-based expression. B) The fusion product of the coding sequences of IRES and LUC were cloned downstream of the T7 promoter in the EasyXpress pIX3.0 expression vector in order to obtain IRES-based expression constructs. As an example, C) illustrates the EasyXpress pIX3.0 vector backbone equipped with the CrPV IGR IRES and a gene of interest. Restriction sites are shown for cloning purposes.
