# Supplementary material for: IRES-Mediated Translation of Membrane Proteins and Glycoproteins in Eukaryotic Cell-Free Systems
Source: PLoS One. 2013 Dec 20;8(12):e82234. doi: 10.1371/journal.pone.0082234 (PMC3869664; doi:10.1371/journal.pone.0082234)
Supplement: Figure S3 — Workflow of the cell-free protein synthesis reactions using eukaryotic cell extracts. (DOCX) [file pone.0082234.s003.docx]

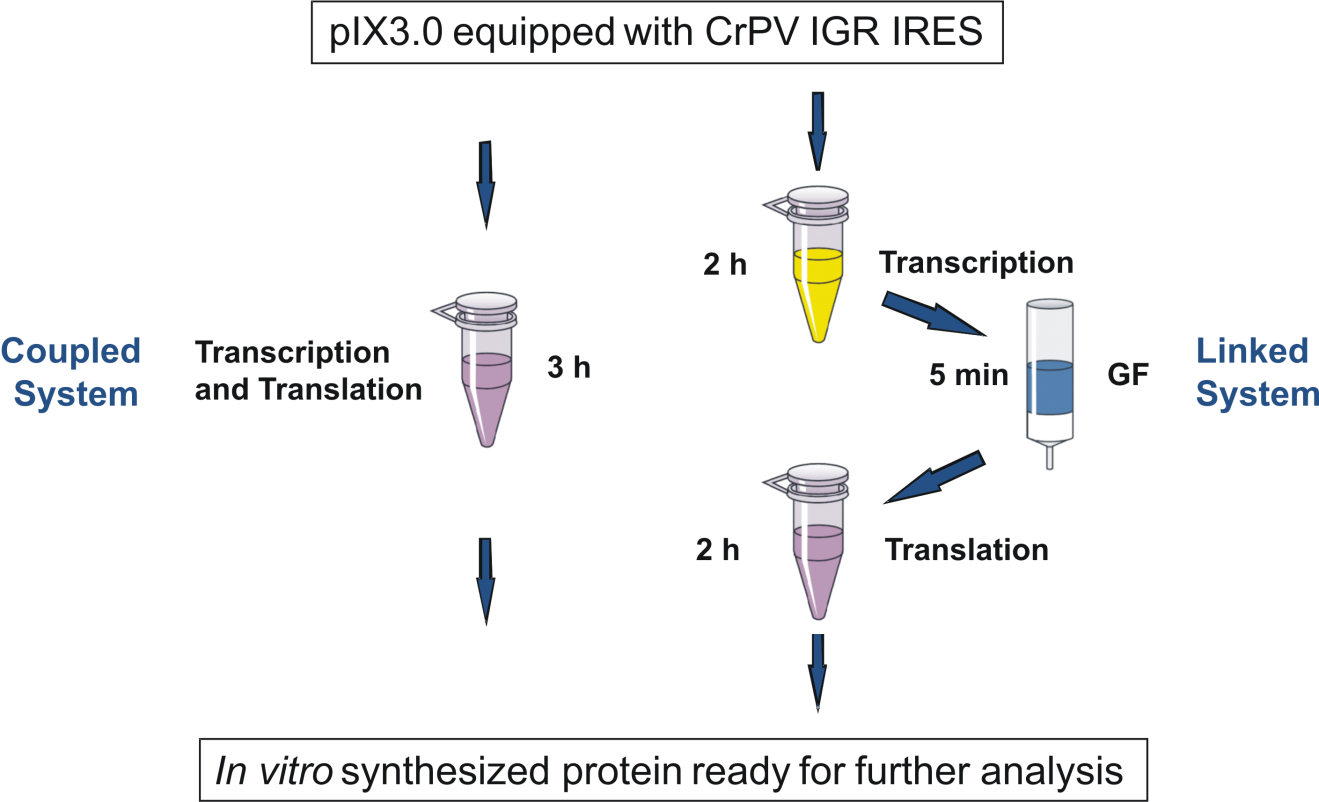


Figure S3. Workflow of the cell‐free protein synthesis reactions using eukaryotic cell extracts. The coupled system combines transcription and translation in a single batch reaction. Alternatively, protein expression can be performed in a linked system where transcription and translation reactions are separated by an intermediate gel filtration (GF) step.
