# Supplementary material for: IRES-Mediated Translation of Membrane Proteins and Glycoproteins in Eukaryotic Cell-Free Systems
Source: PLoS One. 2013 Dec 20;8(12):e82234. doi: 10.1371/journal.pone.0082234 (PMC3869664; doi:10.1371/journal.pone.0082234)
Supplement: Figure S4 — Impact of the cap structure on the expression levels in coupled eukaryotic cell-free systems. (DOCX) [file pone.0082234.s004.docx]

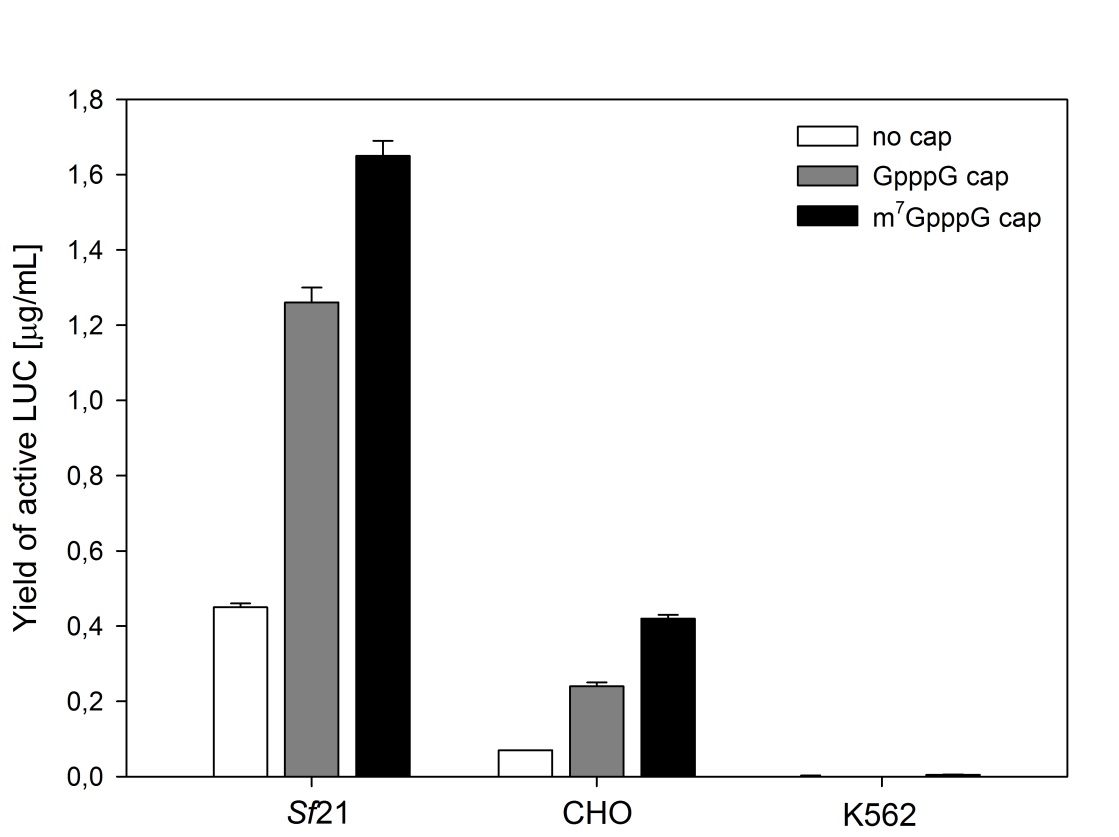


Figure S4. Impact of the cap structure on the expression levels in coupled eukaryotic cell-free systems. Cell-free protein synthesis was performed in the absence or presence of cap structures (GpppG, m^7^GpppG) at standard conditions. Relative light units were measured using a LUC reporter assay and the yields of active LUC in µg/mL were calculated based on a calibration curve. Yields of active LUC were determined from three independent experiments and the corresponding standard deviations were calculated.
