# Supplementary material for: IRES-Mediated Translation of Membrane Proteins and Glycoproteins in Eukaryotic Cell-Free Systems
Source: PLoS One. 2013 Dec 20;8(12):e82234. doi: 10.1371/journal.pone.0082234 (PMC3869664; doi:10.1371/journal.pone.0082234)
Supplement: Figure S5 — Evaluation of optimal KOAc concentration in the wheat germ-based CECF system. (DOCX) [file pone.0082234.s005.docx]

Figure S5. Evaluation of optimal KOAc concentration in the wheat germ-based CECF system. *De novo* synthesized LUC was monitored after 24 h of incubation at 24°C. Cell-free protein synthesis was performed using the optimized vector equipped with the CrPV IGR IRES harboring an AUG-to-GCU mutation of the initiation codon in the EasyXpress pIX3.0 vector backbone. Yields of active LUC were determined from three independent experiments using a LUC reporter assay and the corresponding standard deviations were calculated. Protein yields were normalized to the reaction with the highest yield of active LUC (= 100%).
