# Supplementary material for: IRES-Mediated Translation of Membrane Proteins and Glycoproteins in Eukaryotic Cell-Free Systems
Source: PLoS One. 2013 Dec 20;8(12):e82234. doi: 10.1371/journal.pone.0082234 (PMC3869664; doi:10.1371/journal.pone.0082234)
Supplement: Figure S6 — Integration of membrane proteins into microsomal vesicles. (DOCX) [file pone.0082234.s006.docx]

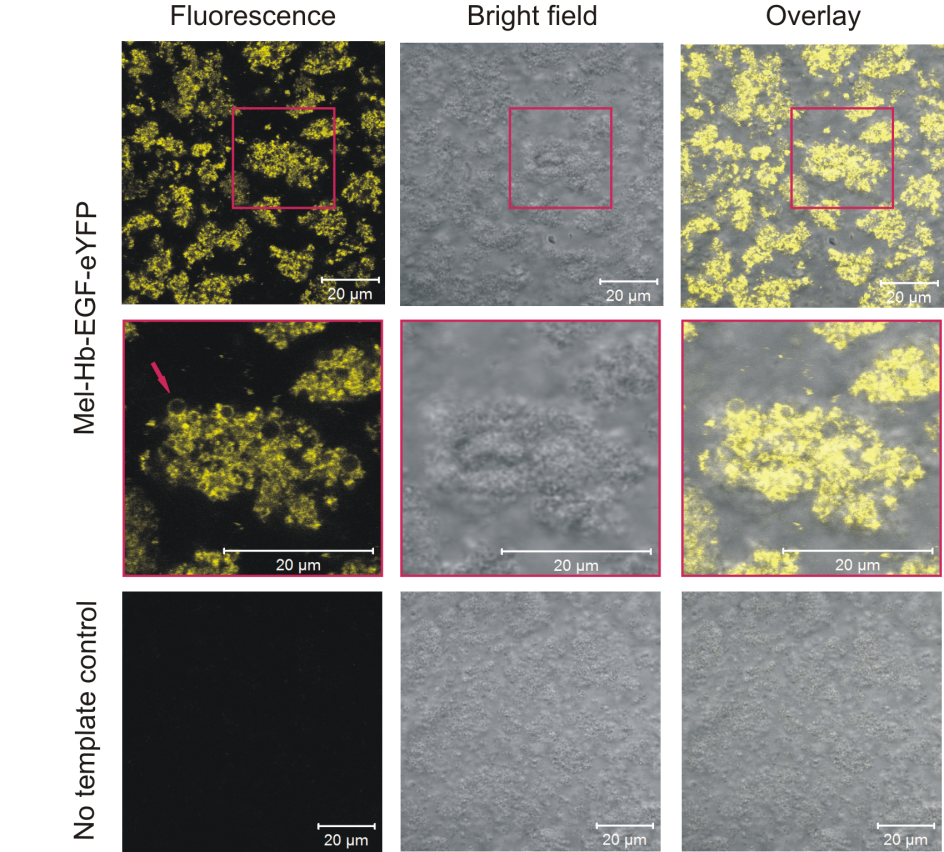


Figure S6. Integration of membrane proteins into microsomal vesicles. CLSM images of Mel-Hb-EGF-eYFP synthesized in *Sf*21 cell lysate using the EasyXpress pIX3.0 vector backbone equipped with the CrPV IGR IRES (GCT) as DNA template. Insect microsomes with a fluorescent membrane have been visualized by CLSM, indicating the insertion of *de novo* synthesized Mel-Hb-EGF-eYFP into the vesicular membrane. The image depicts a detailed version of the picture which is shown in Figure 5. Samples were excited at 488 nm and fluorescence emission was recorded with a long-pass filter in the wavelength range above 505 nm (LSM 510 Meta microscope, Zeiss).
