# Supplementary material for: IRES-Mediated Translation of Membrane Proteins and Glycoproteins in Eukaryotic Cell-Free Systems
Source: PLoS One. 2013 Dec 20;8(12):e82234. doi: 10.1371/journal.pone.0082234 (PMC3869664; doi:10.1371/journal.pone.0082234)
Supplement: Table S1 — Primer sequences used for DNA template design. (DOCX) [file pone.0082234.s007.docx]

Table S1. Primer sequences used for DNA template design.

| **Name** | **Oligonucleotide sequence** |
| --- | --- |
| **CrPV-F** | 5' TTA AGA AGG AGA TAA ACA AAA GCA AAA ATG TGA TCT 3' |
| **CrPV (GCT)-oe-EPO-R** | 5' AGG ACA TTC GTG CAC CCC AGC AGG TAA ATT TCT TAG GT 3' |
| **CrPV (ATG)-oe-LUC-R** | 5' TGT TTT TGG CGT CTT CCA TAG GTA AAT TTC TTA G 3' |
| **CrPV (GCT)-oe-LUC-R** | 5' TGT TTT TGG CGT CTT CAG CAG GTA AAT TTC TTA G 3' |
| **CrPV (GCT)-oe-Mel-R** | 5' AAC GTT GAC TAA GAA TTT AGC AGG TAA ATT TCT TAG GT 3' |
| **EMCV-F** | 5' TTA AGA AGG AGA TAA ACA CCC CCC CCT AAC GTT ACT 3' |
| **EMCV-oe-LUC-R** | 5' TGT TTT TGG CGT CTT CCA TAT TAT CAT CGT GTT TTT CA 3' |
| **LUC-F** | 5' ATG GAA GAC GCC AAA AAC ATA 3' |
| **LUC-R** | 5' CTT GGT TAG TTA GTT ATT ACA CGG CGA TCT TTC CGC CCT 3' |
| **RhPV-F** | 5' TTA AGA AGG AGA TAA ACA GAT AAA AGA ACC TAT AAT 3' |
| **RhPV-oe-LUC-R** | 5' TGT TTT TGG CGT CTT CCA TTA TAA ATA GAT AAA GCT A 3' |
| **RS 3'** | 5' TAA TAA CTA ACT AAC CAA GAT CTG TAC CCC TTG GGG CCT CTA AAC GGG TCT TGA   GGG GTT TTT TGG ATC CGA ATT CAC CGG TGA TAT CAT 3' |
| **RS 5'** | 5' ATG ATA TCT CGA GCG GCC GCT AGC TAA TAC GAC TCA CTA TAG GGA GAC CAC AAC  GGT TTC CCT CTA GAA ATA ATT TTG TTT AAC TTT AAG AAG GAG ATA AAC AAT G 3' |
