# Supplementary material for: IRES-Mediated Translation of Membrane Proteins and Glycoproteins in Eukaryotic Cell-Free Systems
Source: PLoS One. 2013 Dec 20;8(12):e82234. doi: 10.1371/journal.pone.0082234 (PMC3869664; doi:10.1371/journal.pone.0082234)
Supplement: Table S2 — Expression vectors used in this study. (DOCX) [file pone.0082234.s008.docx]

Table S2. Expression vectors used in this study.

| **Expression vector** | **Target protein** | **Size [bp]** |
| --- | --- | --- |
| pIX2.0-Mel-EPO | EPO | 3811 |
| pIX3.0-CrPV IGR IRES (GCT)-EPO | EPO^1^ | 3591 |
| pIX3.0-CrPV IGR IRES (ATG)-LUC | LUC | 4662 |
| pIX3.0-CrPV IGR IRES (GCT)-LUC | LUC^1^ | 4662 |
| pIX3.0-CrPV IGR IRES (GCT)-Mel-EGFR-eYFP | Mel-EGFR-eYFP^1^ | 7374 |
| pIX3.0-CrPV IGR IRES (GCT)-Mel-eYFP | Mel-eYFP^1^ | 3792 |
| pIX3.0-CrPV IGR IRES (GCT)-Mel-Hb-EGF-eYFP | Mel-Hb-EGF-eYFP^1^ | 4386 |
| pIX3.0-EMCV IRES-LUC | LUC | 5031 |
| pIX3.0-LUC | LUC | 4470 |
| pIX3.0-Mel-EGFR-eYFP | Mel-EGFR-eYFP | 7182 |
| pIX3.0-Mel-eYFP | Mel-eYFP | 3603 |
| pIX3.0-Mel-Hb-EGF-eYFP | Mel-Hb-EGF-eYFP | 4197 |
| pIX3.0-RhPV 5' IRES-LUC | LUC | 5048 |
| ^1^ The first translated amino acid is alanine encoded by a GCU base sequence. | | |
